# Simulation Models for Socioeconomic Inequalities in Health: A Systematic Review

**DOI:** 10.3390/ijerph10115750

**Published:** 2013-11-04

**Authors:** Niko Speybroeck, Carine Van Malderen, Sam Harper, Birgit Müller, Brecht Devleesschauwer

**Affiliations:** 1 Institute of Health and Society (IRSS), Université Catholique de Louvain, Brussels 1200, Belgium; E-Mails: carine.vanmalderen@uclouvain.be (C.M.); Brecht.Devleesschauwer@UGent.be (B.D.); 2 Department of Epidemiology, Biostatistics & Occupational Health, McGill University, Montreal, QC H3A0G4, Canada; E-Mail: sam.harper@mcgill.ca; 3 Department Ecological Modelling, Helmholtz Centre for Environmental Research—UFZ, Leipzig 04318, Germany; E-Mail: birgit.mueller@ufz.de; 4 Department of Virology, Parasitology and Immunology, Faculty of Veterinary Medicine, Ghent University, Ghent 9000, Belgium

**Keywords:** models, simulations, socioeconomic, health

## Abstract

*Background*: The emergence and evolution of socioeconomic inequalities in health involves multiple factors interacting with each other at different levels. Simulation models are suitable for studying such complex and dynamic systems and have the ability to test the impact of policy interventions *in silico*. *Objective*: To explore how simulation models were used in the field of socioeconomic inequalities in health. *Methods*: An electronic search of studies assessing socioeconomic inequalities in health using a simulation model was conducted. Characteristics of the simulation models were extracted and distinct simulation approaches were identified. As an illustration, a simple agent-based model of the emergence of socioeconomic differences in alcohol abuse was developed. *Results*: We found 61 studies published between 1989 and 2013. Ten different simulation approaches were identified. The agent-based model illustration showed that multilevel, reciprocal and indirect effects of social determinants on health can be modeled flexibly. *Discussion and Conclusions*: Based on the review, we discuss the utility of using simulation models for studying health inequalities, and refer to good modeling practices for developing such models. The review and the simulation model example suggest that the use of simulation models may enhance the understanding and debate about existing and new socioeconomic inequalities of health frameworks.

## 1. Introduction

Socioeconomic status (SES) has traditionally been defined by relevant PROGRESS factors, *i.e.*, Place of Residence, Race/ethnicity, Occupation, Gender, Religion/culture, Education, Socioeconomic status, Social capital/networks [1]. An association between SES and health has been demonstrated in numerous studies [2], resulting in the so-called socioeconomic gradient in health. 

Moreover, when these health inequalities are quantified by the concentration index [3] as an indicator, they can be “unpacked” through a decomposition analysis [4]. Such an analysis provides interesting insights on the contribution of different determinants to socioeconomic health inequality (e.g., quantifying the importance of illiteracy among women on child health inequalities) [5,6,7,8,9]. However, a decomposition analysis is based on a generalized linear model [4] and may therefore suffer from limitations inherent to such a model. 

Generalized linear models are appropriate for identifying isolated relationships between covariates and health while taking into account potential confounders. However, interrelations among individuals can lead to violations of the stable unit treatment value assumption, since e.g., an education intervention affecting the health condition of one individual could also affect the health condition of his/her friend. A further limitation is that in these models all variables are dealt with at the same level (*i.e.*, additively, as explanatory variables at the right side of the linear equation), whether endogenous (such as genes), individual-level (such as age, education, or an individual behavior), neighborhood-level (such as the suitability of the environment), school-level (such as availability of health education), policy-level, and so forth. An analysis of socioeconomic health inequalities should embrace the multi-level aspect of the different determinants. 

Multilevel, or hierarchical, regression models can consider the contribution of factors at multiple levels, but do little to deal with a fundamental limitation of all generalized linear models, namely that these models hardly take into account the dynamic, reciprocal, discontinuous or changing relations between exposures and outcomes [10]. In alcohol consumption, for example, individual socioeconomic position contributes to the type of neighborhood a person can afford to live in and to the level of alcohol consumed. But individual socioeconomic position is also a product of the types of income-generating opportunities afforded by the neighborhood socioeconomic environment [11]. 

In studies on socioeconomic inequalities in health it may also be important to incorporate complex and indirect health effects for a better understanding of causal pathways. Nandi *et al*. show an example of how early exposure to a poor socioeconomic environment may impact health in later life is [12]. In their example, the methodological challenge is twofold: first, early life SES is associated with later life SES, and second, and more challenging methodologically, early life SES may lead to behaviors adopted (e.g., smoking, poor diet) that impact SES in later life. Although structural equation models can assist in understanding causal pathways, more complex models may be needed for estimating relations between variables in a dynamic process that produces health inequality over time. Simulation models, offering simplified representations of a certain real-life system [10,13,14], have the potential to fulfill this need. Simulation models can be specified in many different ways, and the various existing simulation approaches may deal with different aspects of a complex system. 

By identifying the mechanisms responsible for the generation and maintenance of health inequalities, simulation models can be used as a tool for identifying new options for policy interventions. Furthermore, once a simulation model is established, it can be used as a virtual lab to assess the effects of specific interventions. Indeed, complex systems modeling approaches have the potential to integrate the growing knowledge about multilevel causes of health and their patterns of feedback and interaction, and to inform how specific policy interventions could influence the health of populations [10]. This paper provides a systematic review on the use of simulation models developed to better understand or modify socioeconomic inequalities in health. Using a simple agent-based model (ABM), we show how simulation models can be developed and used to study socioeconomic inequalities in health.

## 2. Experimental Section

### 2.1. Systematic Review

The systematic review followed the reporting guidelines of PRISMA [15] and PRISMA-Equity 2012 [16]. However, as the review focused on a qualitative synthesis of the simulation models (and not their results), several items in the PRISMA statement (e.g., “summary measures”) were not applicable in our review. 

#### 2.1.1. Eligibility Criteria

Studies with the following characteristics were eligible: the target population is human individuals or groups; the intervention or exposure involves a socioeconomic factor; the outcome variable is a health status, behavior or access to health care; and the study design is a simulation model. No restrictions were applied on the year, language, type or status of the publication.

#### 2.1.2. Information Sources and Search

Electronic searches were conducted using PubMed, Scopus and the Web of Knowledge on 22 January 2013. The following terms and operators were used and applied on title/abstract/keywords ((tw) in PubMed): (“simulat*” OR “equation based-model*” OR “process-based model*” OR “dynamic model*” OR “multi-agent*” OR “differential equation*” OR “compartmental model*” OR “difference equation*” OR “projection model*” OR “systems analysis” OR “systems model*” OR “computer model*” OR “agent based” OR “individual based” OR “rule based” OR “mathematical model*” OR “microsimulation”) AND (polarization OR polarisation OR imparit* OR parit* OR unjustness OR discrimination OR inequalit* OR disparit* OR equit* OR inequit* OR equalit*) AND (sickness OR sanity OR medical OR health OR healthy* OR healthi* OR illness* OR disabilit* OR morbidit* OR mortalit* OR disease OR diseases). The search terms were discussed and approved by four of the authors based on their expertise in simulation models or in health inequalities research. The search strategy was tested and fine-tuned in Scopus. Records were imported into Reference Manager (Thomson Reuters Professional Edition version 12) and duplicates, defined as records with similarity in titles >87% (default parameters in Reference Manager) and the same publication date, were removed. The remaining duplicates identified by progressively decreasing the degree of similarity between titles and not using the publication date criterion were manually removed. 

#### 2.1.3. Study Selection

Titles (and abstracts if necessary) were screened for eligibility. As the number of eligible studies was greater than expected, selection criteria were refined to better meet the aim of the review. Studies assessing socioeconomic inequalities in health using a simulation model were selected if the following criteria were met: (1) the study aims to better understand or modify a difference in health (health status, health behavior, access to health care or exposition to a health-threatening exposure) between socioeconomic (PROGRESS) sub-groups of the population; and (2) the method used is a simulation model, defined as an experiment performed on a representation of a system. Finally, only full research articles published in English were selected. 

#### 2.1.4. Data Collection Process and Data Items

The aim of the study, the type and features of the simulation model, the structural determinant(s), the health outcome(s), the country, the target population, the main findings, authors and publication dates were extracted into a pre-designed form. The number of studies by characteristic was counted and plotted using R version 3.0.1 [17].

Simulation models were first classified in two classes according to the level of experimentation. “Individual-based simulation models” perform simulation experiments at individual level (e.g., individuals’ attributes, behaviors or relationships). “Population-based simulation models” perform simulation experiments at population level (e.g., state processes and transition probabilities, components or dynamics). In the individual-based simulation models group, three different approaches were identified: microsimulation, agent-based and network. In the population-based simulation models group, seven different approaches were identified: state-transition, optimization, risk assessment, projection, game, behavioral/stress and diffusion. The description of the different simulation approaches is presented in Table 1. 

Socioeconomic determinants were categorized into: place of residence, race/ethnicity, occupation, gender, socio-cultural factors, education, economic status, social capital, insurance coverage, marital status and housing. Health outcomes were categorized into health status, life expectancy, mortality, child health, mental health, obesity, infectious disease, cancer, health behavior, access to health care/treatment/prevention and environmental exposition. These categorizations helped in identifying the main situations of inequality studied and the related simulation approach used.

**Table 1:** Description of simulation model approaches.

**Individual-based**	
Microsimulation	In these models, individuals are represented as passive micro-level entities. The experiment consists in modifying individuals’ attributes. Analyses are made using regression-based or econometric methods.
Agent-based	In agent-based models, individuals are represented as active (*i.e.*, are able to adapt to the environment, interact with others and make autonomous decisions) micro-level entities. The experiment consists in modifying agents’ rules or the system structure.
Network	In network models, individuals are represented as micro-level entities interacting with each other. The experiment consists in modifying individuals’ relationships.
**Population-based**	
State-transition	State-transition models are developed with differential equations. The population is divided in subgroups through which individuals pass. These subgroups may be defined according to health states or by SES. This category includes system dynamics models with stocks, flows and feed-back loops, epidemic models (e.g., Susceptible/Infected/Recovered models), and Markov models.
Optimization	In this category, the basic components modeled are facilities or services. The optimal allocation of health care resources is estimated by maximizing or minimizing a function.
Risk assessment	In these models, the unequal distribution of a health risk of a simulated exposure is estimated.
Projection	Based on actual population data and rates, these models project future population demographics under several assumptions.
Game	These models study strategies in which the decision of an individual or group depends on the decision of the others.
Behavioral/stress	Behavioral: the model consists in a recursive system of equations. In this model, individuals maximize a lifetime utility function. Stress: individual’s health is determined by endowments, permanent shocks, and transitory shocks.
Diffusion	Temporal and spatial diffusion of an innovation are modeled as subsystems transitions from dynamic to steady states.

The description of simulation model approaches was based on the studies included in the review.

Several characteristics of the systems modeled in the studies were extracted as described by the following keywords: (1) multilevel—the system components may be aggregated at distinct levels (e.g., endogenous, individual, network, neighborhood), (2) dynamic—the system evolves over time; the relations between some elements of the system depend on time, (3) stochastic—the system includes an element of random nature or an element that can be specified only probabilistically, (4) heterogeneous individuals—differentiated (with at least two attributes) individuals are represented as micro-level entities and, if active, are able to interact with each other or to adapt to their environment, (5) feedback loop—the system includes a chain of causes and effects that forms a loop, and (6) spatial—the system has a spatial dimension; the relations between some elements of the system depend on space. 

Finally, information about the model validation and utilization was extracted. The method section of the studies was screened for validation methods. Whether or not the model aimed to develop a framework or to test an intervention or scenario was extracted.

### 2.2. Agent-Based Model (ABM)

**Figure 1:**
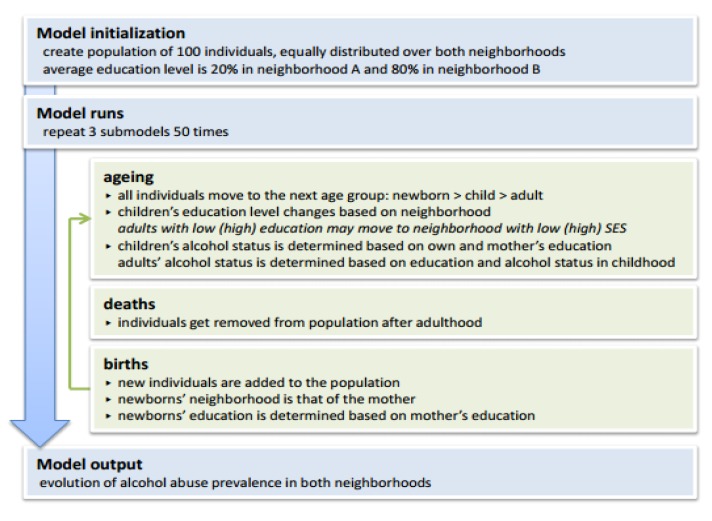
Schematic representation of the agent-based simulation model of alcohol abuse in two neighborhoods with distinct socioeconomic levels.

To illustrate the use of simulation models for studying socioeconomic inequalities of health, a simple ABM aiming to study how socioeconomic differences in alcohol abuse may emerge was developed. Figure 1 shows a schematic representation of this model.

The model simulates the life course of individual women, who are born, age, give birth, and die (the system represented is dynamic). Two neighborhoods are defined, reflecting low and high SES (the system is multilevel); however, the model is not spatially explicit, as the distance between neighborhoods is not explicitly modeled. The model consists of heterogeneous individuals, who interact with each other and their environment. The attributes of the individuals may change over time, based on probabilistic processes. Each individual has an education level that depends on the mother’s education level, but can change based on the neighborhood. Indeed, the model assumes that children may increase or decrease their education level based on the average education level in their neighborhood. Individuals are further allowed to develop alcohol abuse depending on prior alcohol abuse and on the education level of the individual and its mother. Alcohol abuse in childhood is assumed to depend on the individual’s education level and that of its mother, while alcohol abuse in adulthood is assumed to depend on the individual’s education level and alcohol abuse during childhood. This situation represents the baseline model. In a next scenario, we allow individuals to change neighborhood based on their education level. Individuals with a high education will move with a certain probability to a high SES neighborhood, and *vice versa*. As a result, we thus create a feedback loop between education level and neighborhood. Indeed, the education level in childhood is assumed to depend on the neighborhood, while the neighborhood in adulthood is assumed to depend on education level. 

Validation of the model only occurred through expert judgments of the input parameters and simulated outputs. If the model were to be used for more in-depth research, rather than as an example of the use of ABMs, model validation through comparison with observed alcohol abuse patterns would be essential. Table A1 provides a more detailed description of the ABM, following the Overview, Design concepts and Details (ODD) protocol proposed by Grimm *et al*. [18]. The model was developed and run in R version 3.0.1 [17], and the model’s source code is provided in Table A1 and Algorithm A1.

## 3. Results

### 3.1. Review

#### 3.1.1. Description of Selected Studies

**Figure 2:**
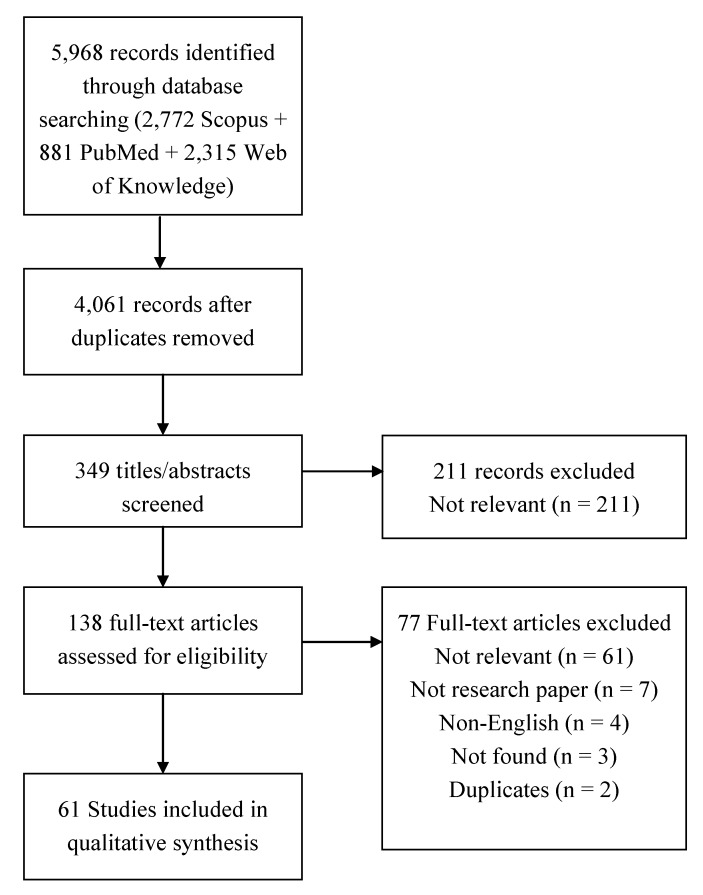
Flow of information through the different phases of the review.

The 61 studies selected [19,20,21,22,23,24,25,26,27,28,29,30,31,32,33,34,35,36,37,38,39,40,41,42,43,44,45,46,47,48,49,50,51,52,53,54,55,56,57,58,59,60,61,62,63,64,65,66,67,68,69,70,71,72,73,74,75,76,77,78,79] were published between 1989 and 2013 (Figure 2). They were conducted in all continents: America (n = 28), Europe (n = 16), Asia (n = 10), Africa (n = 5) and Australia (n = 3) (Table A2). The review of the simulation models identified 16 individual-based models and 45 population-based models. The different approaches are summarized in Table 2. 

**Table 2:** Number of studies reporting several features of simulation models in total and by model type.

		Individual-based			Population-based						
	Total	Microsimulation	Agent-based	Network	State.transition	Optimization	Risk assessment	Projection	Game	Behavioral	Diffusion
**Total number of studies**	61	11	4	1	21	13	4	2	2	2	1
**Characteristics of the system modeled**											
1. Multilevel	59	10	4	1	20	13	4	2	2	2	1
2. Dynamic	40	6	4	1	20	2	2	1	1	2	1
3. Stochastic	34	6	4	1	13	4	3	0	1	2	0
4. Heterogeneous micro-level entities	40	11	4	1	13	3	2	2	1	2	1
interacting with each other	6	0	2	1	2	0	0	0	1	0	0
adapting to their environment	10	1	3	0	3	1	0	0	1	1	0
5. Feed-back loop	7	0	2	0	5	0	0	0	0	0	0
6. Spatial	37	6	4	0	6	13	4	1	1	1	1
**Validation and utilization of the model**											
Validation on observational data	14	2	1	0	6	4	1	0	0	0	0
Development of a framework	17	1	1	0	3	8	2	1	0	1	0
Test of an intervention/scenario	48	5	4	1	18	13	3	2	2	0	0

**Figure 3:**
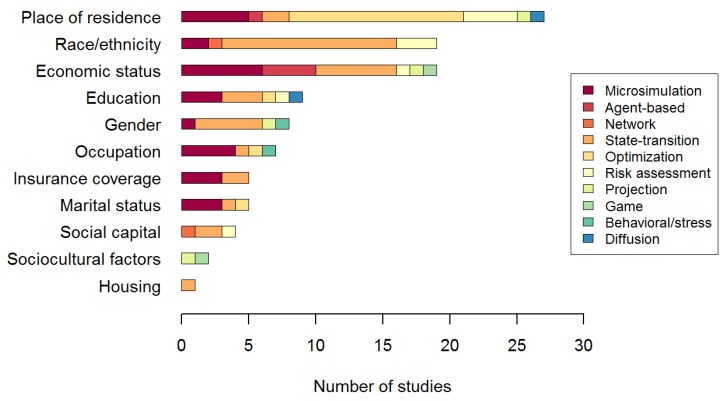
Structural determinants included in the selected studies.

**Figure 4:**
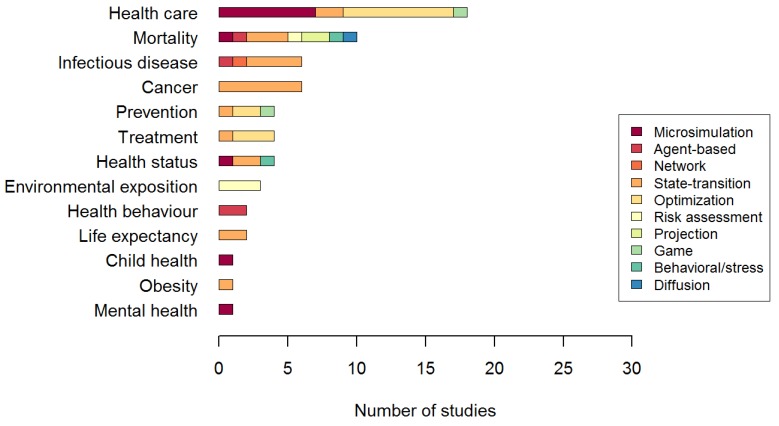
Health outcomes included in the selected studies.

The most common simulation approaches were state-transition models, optimization models and microsimulations. In several studies [39,40,43,45,47,49,50], state-transition models were used with a microsimulation. In one study [38], state-transition and network approaches were combined. To facilitate the description, these studies were classified as state-transition models as this was considered as the main approach of the study. All PROGRESS factors were represented in the selected set of publications. The determinants reported were mostly place of residence, race/ethnicity and economic status (Figure 3). The health outcomes modeled are shown in Figure 4. Inequalities in health status (self-reported, nutritional status, disease, mortality, life expectancy, preterm birth) were modeled in 31 studies. Unequal access to health care (health facilities, treatment or prevention) was modeled in 27 studies. The remaining studies modeled inequalities in an environmental exposure (n = 3) [70,71,72] and inequalities in health behavior (n = 2) [30,31]. 

#### 3.1.2. Characteristics of the System Modeled

In the selected studies the use of simulation models allowed for a better understanding the complex dynamics giving rise to health inequalities. The system encompassing a health inequality may be multilevel, dynamic, spatial stochastic, and includes active heterogeneous micro-level entities or feed-back loops. These complex features were appropriately taken into account through the various simulation modeling approaches (Table 2), as illustrated hereafter. 

The complexity of the relationships between the components of the system was present in all studies to varying degrees. Complexity was high in a study assessing the causal pathways of the multiple social and biological determinants of health in the city of Toronto [48]. In this simulation model, many determinants interact with direct or indirect impacts on health, strong or weak causal effects and time delays. 

Nearly all reported models included more than one level of factors, e.g., cold-ischemia time of an organ transplant (endogenous), waiting time of the patient (individual), location of the health center (neighborhood) and allocation rules (policy) [57]. 

Two-thirds of the models were dynamic. The time dimension was especially essential when outcomes such as inequalities in future disease incidence (e.g., state-transition models) or life trajectories (e.g., behavioral/stress model) were studied. 

Stochasticity was introduced in the models in several ways and for various reasons. In a spatial stochastic multimedia exposure model [69], probability density distributions of random model input variables were used to compute exposure and risk indicators. In a spatial interaction study [60], random fluctuations were introduced in the data to test the robustness of the model. In the network simulation of HIV transmission [34], every contact (relationship) was made with a randomly chosen member of the population. 

Individuals were represented as micro-level entities in two-third of studies. In 16 studies, individuals were active, either able to interact with others or to adapt to their environment. Modeling individual interactions was essential in a study on influenza vaccination and transmission [32]. Indeed, this study emphasized that poorer counties tend to have high-density populations and more children and other higher-risk people per household, resulting in more interactions and both increased transmission of influenza and greater risk for worse influenza outcomes. In this simulation, virtual people moved throughout a region in patterns similar to those actually observed in real life, interacting with each other at places such as offices and schools, based on the day of the week and each person’s characteristics. Ten papers modeled an adaptive behavior between people and their environment over time. For example, Auchincloss et al. [30] assessed income inequalities in diet in the context of residential segregation. In this study, the selection of a food store by the household was determined by the price of food, the distance to the store, its habitual behavior and the preference for healthy food. 

A feed-back loop was modeled in seven papers, mainly agent-based or state-transition models. As an example, a study found that feedbacks between disease ecology and economics can create clusters of low income and high disease that can stably persist in populations that become otherwise predominantly rich and free of disease [38]. 

The spatial dimension was introduced in the model as observable geographical units (region, county, census output area…) in most studies and in all optimization and risk assessment models.An artificial space was simulated in the four agent-based models (grid space) and in two other studies (Banach space) [44,75]. 

#### 3.1.3. Validation and Utilization of the Model

Among the 16 studies having reported a validation process in their methods, most (n = 14) compared predicted results with observational data (*i.e.*, pattern-oriented modeling; discussed in more detail below) and two compared model results to experts’ opinions [37,48]. 

In 17 studies, mainly for optimization models, an explicit aim was to provide a conceptual framework of the studied phenomenon. 

If validated, the simulation model may then be used as a tool to test the effect of a virtual intervention. Most studies tested the impact of several scenarios/interventions on inequalities: allocation policies, health reform strategies, treatment or prevention programs, relocation of facilities *etc*. Some studies used existing simulation models. The MISCAN model projects US cancer population trends and was used to test the impact of cancer screening [39,40,45,50]. The Prevent model estimates the health benefits in a population due to changes in risk factor prevalence and was used to test the impact of interventions to prevent smoking [52,54]. The Life Saved Tool projects the reduction in the mortality rates and stunting that could be achieved if the coverage levels of specific interventions were increased on the basis of baseline characteristics, demographic characteristics, and coverage targets. The tool was used to estimate the effects of different intervention packages and coverage levels on under-5 mortality and malnutrition [74].

### 3.2. Agent-Based Illustrative Model

**Figure 5:**
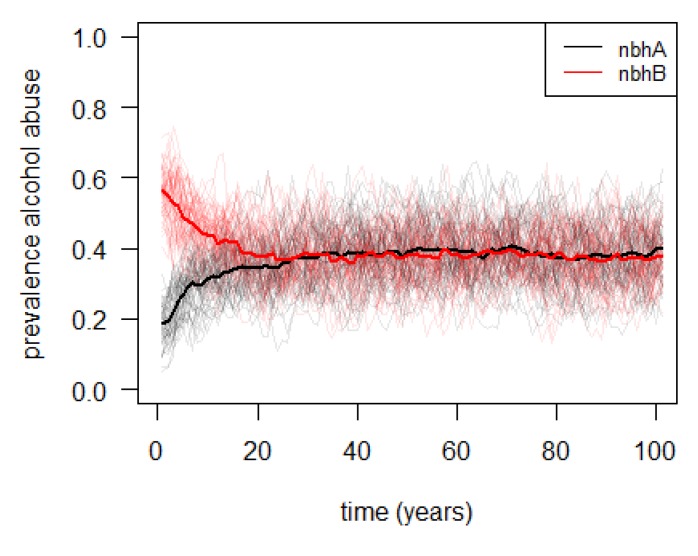
Simulated prevalence of alcohol abuse in two neighborhoods (“nbhA” and “nbhB”, with high, respectively, low, socioeconomic status), assuming no education-dependent mobility between neighborhoods; the thin lines (highly variable) represent the output of 100 individual model runs, while the thick lines represent the averages of all individual model runs.

**Figure 6:**
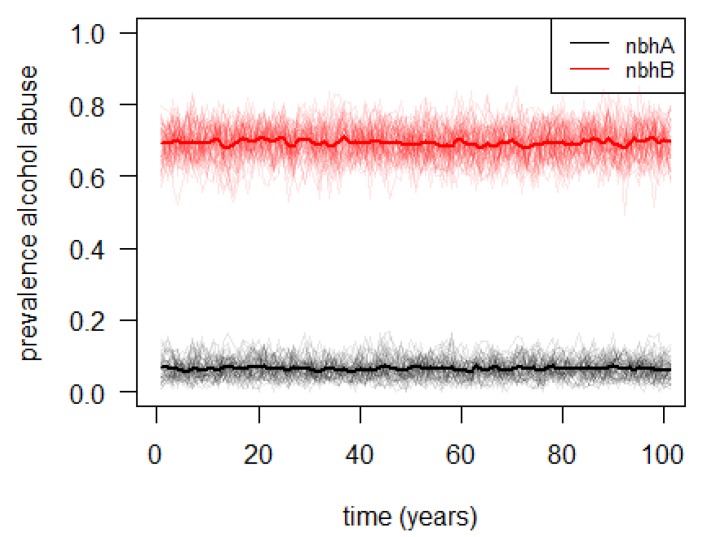
Simulated prevalence of alcohol abuse in two neighborhoods (“nbhA” and “nbhB”, with low, respectively, high, socioeconomic status), assuming education-dependent moving between neighborhoods; the thin lines represent the output of 100 individual model runs, while the thick lines represent the averages of all individual model runs.

Figure 5 and Figure 6 show the simulated level of alcohol abuse in both neighborhoods, for the baseline and the extended model. 

In the baseline model, where no education-dependent moving between neighborhoods was assumed, the alcohol abuse prevalence in both neighborhoods evolves from the original state to a similar state. In other words, in this situation no inequalities are observed between the two neighborhoods. In the extended model, however, a clear qualitative difference between both neighborhoods emerges, reflecting clear inequalities between the two neighborhoods. This observation suggests that, subject to the model assumptions, mobility may be a driving force behind socioeconomic health inequalities. Note that the quantitative differences are the result of arbitrary parameter settings, and should thus not be interpreted directly.

## 4. Discussion and Conclusions

Determinants of health shape health inequalities in complex ways, requiring innovative approaches such as the use of simulations. Data, mostly collected survey data, allow the identification of gaps in health between socio-economic groups, and can in addition be analyzed with traditional statistical techniques such as generalized linear models, revealing relationships between observed health inequalities and their determinants. Such analyses can be indicative of health gaps and important determinants, but may not reveal the mechanisms driving socio-economic inequalities of health. The detection of such mechanisms requires tools that can account for feedbacks, interrelations among agents (e.g., humans and the environment) and discontinuous non-linear relations.

Through a systematic literature review, we explored how simulation models have been used so far to study health inequalities. The review shows that simulation models of health inequalities were used in several areas such as health systems research, epidemiology, environmental health or demography. The 61 studies selected used many different types of simulation models. This review sought to identify the main simulation approaches used. The choice of the most appropriate simulation approach should depend on the aim of the study and on the characteristics of the system being modeled. Table 3 summarizes the main situations of inequality modeled in the studies, linking them with the characteristics of the systems modeled and the simulation approach used. Depending on the situation, a characteristic of the system may be more or less important to model (e.g., modeling a dynamic system with active individuals may be particularly interesting when studying the impact of a policy on health behavior, because this impact depends on individuals’ adaptation and may vary over time). Some studies reported in their limitations that their model lacked dynamic [56,62], stochastic [57,75] or individual heterogeneity [25]. These concerns reflect the different considerations that must be balanced when developing any simulation model: the accuracy of the model, its validity and its applicability.

**Table 3:** Overview of the main situations of inequality modeled, main related characteristics of the system, and approach used.

Situation of inequality	Most frequently reported characteristics of the system	Approach used
Unequal access to health care resources	Static, deterministic, spatial Interdependency of components’ decisions Passive heterogeneous individuals	Optimization Game Microsimulation
Unequal health behavior	Dynamic, stochastic, heterogeneous individuals adapting to their environment	Agent-based
Unequal transmission of a disease or unequal disease stages transitions	Dynamic, stochastic, passive (heterogeneous) individuals Heterogeneous individuals interacting with each other	State-transition (+ microsimulation) Network, agent-based
Unequal environmental exposition/risk	Static, passive (heterogeneous) individuals, spatial Dynamic, spatial diffusion	Risk assessment (+ microsimulation) Diffusion
Unequal health status or mortality	Static, deterministic, passive heterogeneous individuals Dynamic, stochastic	Microsimulation, projection Behavioral

Among all approaches explored in the review, ABM is likely the most suitable tool for studying a complex health inequality situation as it integrates most of the characteristics of a complex system. We illustrated the simulation process through the example of a simple hypothetical agent-based simulation model of alcohol abuse. We showed how such simulation models can incorporate feedback loops and provide insights that may not be obtained through classical statistical data models. Indeed, ABMs translate our understanding of a process into simple computer rules, making it possible to simulate complex interactions and non-linear relations. As extending existing ABMs comes down to adding more rules to the model, generating more detailed models can easily be accomplished. ABMs may therefore serve as “virtual labs”, in which our understanding of the process or the impact of possible intervention measures may be tested *in silico*. In our example, this could mean the evaluation of other factors contributing to alcohol abuse, or the evaluation of intervention strategies aimed at e.g., improving the mothers’ education level. As such, ABMs have the potential to become important tools for guiding policy. However, as all models come with inherent assumptions and uncertainties, the usefulness and limitations of the model results need to be clearly communicated with the policy makers. Indeed, as models merely present a simplified representation of reality, they can never replace reality, nor can they exactly predict future events. Therefore, if simulation models were to be used as policy tools, policy makers and other stakeholders should be involved throughout the modeling process [80].

When developing models, good modeling practices should be followed to enhance confidence in the model’s findings. One prominent good modeling practice is model validation, *i.e.*, the process of testing the realism of the model and its outcomes. We note that models can be validated in several ways, depending on the purpose of the model [81]. Structural validation, *i.e.*, an evaluation of the coherence of the simulation model with theory, is not often done. This seems a logical step since, unlike methods for describing epidemics, no mathematical theory exists for health inequalities. Predictive validation, tested by comparing results produced by the models with observations, may help to assess whether or not the mechanism modeled reflects reality. Comparing the simulated results to an observed pattern appears to provide the best validity check. This so-called pattern-oriented approach [82] therefore requires that the model generates patterns that can be observed in real life. In our ABM example, the generated pattern was alcohol abuse prevalence, which is an observable pattern. Furthermore, pattern-oriented modeling makes it possible to calibrate the model, by fine-tuning the parameters to better reproduce the observed pattern. However, our review showed that simulation models of health inequalities are not always validated. The assessment of the model validation used in the selected studies was not straightforward given the diversity of the types of models included, each having their own validity standards. Nevertheless, it appears that validity was not systematically reported, and a test for predictive validity was found in only 22% of studies. Sometimes data are not available, but this does not have to stop the modeler from checking that the results produced are logical e.g., by comparing model results to experts’ opinions [83], as reported in two studies.

Apart from proper validity checks of the models, good modeling practices also include structured documentation of the models and communication with stakeholders. Grimm *et al*. proposed the Overview, Design concepts and Details (ODD) protocol as a standard protocol for reporting ABMs [18]. This idea is extended as a framework for transparent and comprehensive ecological modeling documentation (TRACE), in which all aspects of the modeling cycle are represented, including model development, model testing and analysis, and model application [80]. Although developed for simulation models in ecology, these good modeling practices also apply to simulation models for health inequalities. A transparent and systematic reporting of models will increase confidence in the usefulness of the results. 

Frameworks used for conceptualizing health inequalities and their determinants have been described in various forms in the past [2,84,85]. These frameworks actually already represent models, namely visual models, or, when described in text form, verbal models. However, none of these frameworks was experimented through a simulation model in the selected studies. Testing frameworks quantitatively may be an opportunity for further research in the field of socioeconomic determinants of health inequalities. Indeed, the further development of such frameworks could be guided by simulation models. The review showed that some (17%) models were already used for developing a new framework. We therefore promote the further use of simulation models in line with developing new frameworks.

The review has several limitations. Firstly, the search was limited to three electronic databases. Moreover, the search strategy contains keywords related to inequality but a simulation study of socioeconomic inequalities not containing the chosen terms in its title/abstract/keywords might have been missed. Secondly, the selection of the studies and data extraction was conducted by a single reviewer, increasing the probability of selection/extraction errors. Finally, there is no standard classification of simulation models to apply to our selection of studies. For the majority of studies, the simulation approach was explicitly reported in the studies, but for several studies (e.g., “risk assessment”) an approach was assigned according to the characteristics found in the model. 

With the complexity surrounding the way determinants shape inequalities in health, simulation models will provide a useful added value to the set of more traditional analytical techniques. Studies with a complex design are needed to explore these mechanisms. Simulation models can guide optimal data collection by testing different designs virtually before conducting the study. Although complex issues such as feedback loops can be accounted for by models such as ABMs, the wider use of such models in teaching and research will convince researchers and policy makers to use the available flexibility even more, by e.g., including adaptive behavior of individuals. Furthermore it will guide the data collection in a more efficient way towards policy making and not merely reporting the existence of inequalities. The list of examples published and referred to in this paper together with the illustrative ABM example may assist researchers to develop their own simulation models in the future. 

## Figures and Tables

**Algorithm A1:**
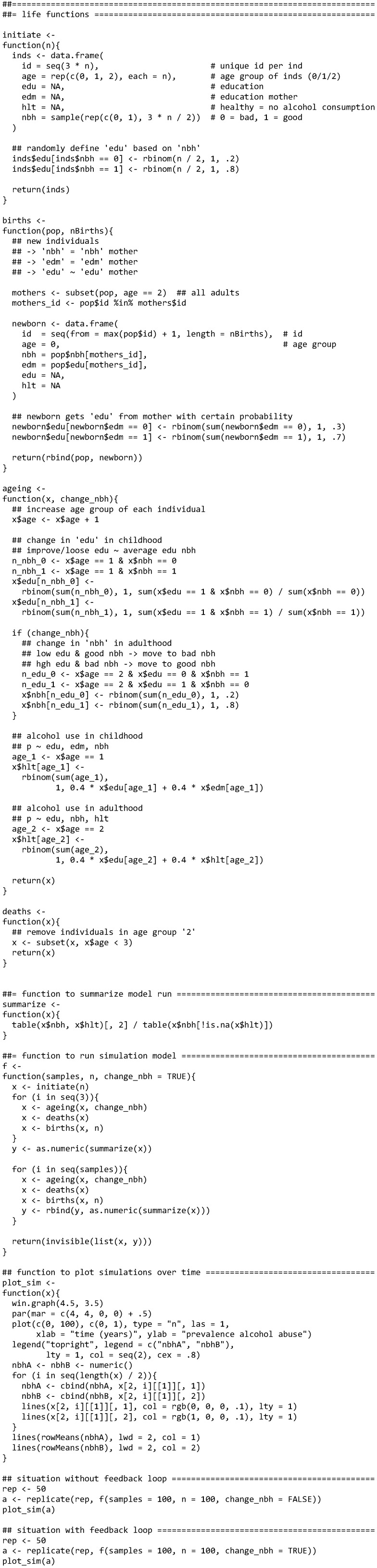
R code for the agent-based model example.

## Appendix

**Table A1:** The following description of the agent-based model for studying socio-economic inequalities in health follows the “ODD” (Overview, Design concepts, and Details) protocol proposed by Grimm *et al*. [18].

Overview	
Purpose	To understand the emergence of socioeconomic health inequalities.
Entities, state variables, and scales	The main model entities are the individual females, each having six state variables: id: unique identification number age: age category (1 = newborn; 2 = child; 3 = adult) edu: own education level (0 = low; 1 = high) edm: mother's education level (0 = low; 1 = high) hlt: own alcohol consumption (0 = no; 1 = yes) nbh: own neighborhood (0 = A; 1 = B) The neighborhood acts as a secondary entity. Its state variables are defined by the inhabitants: average education average alcohol consumption
Process overview and scheduling	The model is updated in discrete time steps: ● **ageing** ○ each individual moves to next age grou ○ children improve or decrease their education level based on the average education level in their neighborhood ○ *adults change neighborhood based on own education level (high edu → nbhA; low edu → nbhB)* ○ alcohol consumption in childhood gets determined based on own and mothers’ education level ○ alcohol consumption in adulthood gets determined based on own education and alcohol use in childhood ● **deaths** ○ individuals who have passed adulthood get removed from the population ● **births** ○ new individuals get added to the population ○ newborns get neighborhood from mother ○ newborns get education from mother with certain probability ● **prevalence assessment** ○ determination of neighborhood-specific average education and alcohol consumption
Design concepts	
Basic principles	The model is based on the ideas that education level depends on the neighborhood and on the mothers’ education level; and that alcohol consumption depends on the own and the mothers’ education level. Optionally, the model can be allowed to assume that adults change neighborhood based on own their education level.
Emergence	The main model results are the neighborhood-specific average education and alcohol consumption levels.
Adaptation	The model contains two adaptive traits: change in education level based on average education level in neighborhood change in neighborhood based on education level
Objectives	The adaptive traits are not linked to any objective.
Learning	There is no change in adaptive traits over time.
Prediction	There are no predictions assumed.
Sensing	The individuals sense the average education level in their neighborhood.
Interaction	There is interaction between mothers and offspring: the newborn gets the neighborhood of the mother the newborn gets the education of the mother with a certain probability
Stochasticity	Mother’s education → newborn’s education: edu ~ Bernoulli(0.70), if edm = 1 edu ~ Bernoulli(0.30), if edm = 0 ∆ Child’s education edu_A_ ~ Bernoulli(*edu*_nbhA_) edu_B_ ~ Bernoulli(*edu*_nbhB_) ∆ Adult’s neighborhood nbh ~ Bernoulli(0.20), if edu = 0 and nbh = 1 nbh ~ Bernoulli(0.80), if edu = 1 and nbh = 0
Collective	Individuals belong to two different neighborhoods; these neighborhoods are entities with own state variables.
Observation	No external data are observed.
Details	
Initialization	The model gets initialized with 100 individuals, equally distributed over both neighborhoods. The initial education level is randomly assigned based on neighborhood: edu_A_ ~ Bernoulli(0.20) edu_B_ ~ Bernoulli(0.80)
Input data	No external input data is used.
Submodels	See R script.

**Table A2:** Description of selected studies.

Name of the model	Socioeconomic determinant(s)	Health outcome(s)	Country	Multilevel	Dynamic	Stochastic	Heterogeneous entities	… interacting	… adapting	Feed-back loop	Spatial	Validated (predictive)	Framework created	Intervention/scenario test	Ref.
**Microsimulation**															
Microsimulation model	Rural/urban, income, employment	Access to GP	Australia	X			X				X			X	[19]
Microsimulation+ decomposition	Household size, income	Number of GP/specialist visits	France	X			X							X	[20]
Microsimulation model	Income, expenditures, taxes	Delivery of health care	UK	X	X	X	X								[21]
Simulation model	Race, education, employment, marital status	Preterm birth, low birth weight, maternal binge drinking	USA			X	X					X		X	[22]
Spatial microsimulation model	Gender, marital status, economic activity, occupational social class	Mental health surveillance	England	X			X				X				[23]
Microsimulation+ decomposition	Household expenditures, education, occupational activity, marital status, insurance coverage, place of residence	Utilization of health services	Palestin	X			X				X				[24]
Discrete simulation model	Ethnicity, insurance	Access to health care	USA	X	X	X	X								[25]
**Microsimulation**															
Spatial microsimulation+ location-allocation model	Census output area	Access to antenatal care	UK	X	X		X				X	X		X	[26]
Roy's model of selectivity	Insurance	Medical utilization	USA	X	X	X	X				X		X		[27]
Microsimulation	Education	Mortality	USA	X	X	X	X							X	[28]
Spatial microsimulation	SES, geographic	Health status	UK	X	X	X	X		X		X				[29]
**Agent-based**															
Agent-based model	Residential segregation	Diet	USA	X	X	X	X		X	X	X	X		X	[30]
Agent-based model	SES	Walking	USA	X	X	X	X	X	X	X	X			X	[31]
Microsimulation model	Salary, income	Influenza vaccination and transmission	USA	X	X	X	X	X			X			X	[32]
Sugarscape model	Wealth	Mortality	(Iran)	X	X	X	X		X				X	X	[33]
**Network**															
Network simulation model	Ethnicity, social network	HIV transmission	USA	X	X	X	X	X						X	[34]
**State-transition**															
Medicare demonstration	Ethnicity, education, public assistance, poverty, unemployment	Primary health care payment	USA	X	X		X				X			X	[35]
	Ethnicity, insurance	Ambulatory health care utilization	US	X	X					X				X	[36]
**State-transition**															
System dynamics model	Insurance	Disease or injury	USA	X	X					X		X	X	X	[37]
Individual-based network model	Poverty	Infectious disease transmission	(USA)	X	X	X	X	X		X			X	X	[38]
State-transition model	Race	Breast cancer outcomes incidence and mortality	USA	X	X	X	X					X		X	[39]
Microsimulation model	Race	Colorectal cancer rate	USA	X	X	X	X					X		X	[40]
Markov state-transition model	Race	Treatment of hypertension, hyperglycemia, hyperlipidemia (cost-effectiveness)	adult	X	X	X								X	[41]
Mathematical transmission model	Health system resources	Mortality from pandemic influenza	Cambodia, Indonesia, Lao PDR, Taiwan, Thailand and Vietnam	X	X	X					X			X	[42]
Markov model + decomposition	Race	Obesity prevalence	USA	X	X	X	X				X				[43]
Transmission model	Gender	HIV/AIDS transmission	African countries	X	X	X					X				[44]
Microsimulation model	Race, gender	Colonoscopic screening	USA	X	X	X	X					X		X	[45]
Simple deterministic mathematical model	Race, gender	Sexually transmitted infections incidence	UK	X			X	X		X		X		X	[46]
Disease simulation model	Race	Cancer control	USA	X	X	X	X						X	X	[47]
System dynamics model	Ethnicity, immigration status, gender, income, housing, social cohesion	Chronic disease, disability, and mortality rate	Canada	X	X				X	X				X	[48]
Discrete-time Markov-chains + microsimulation	Race, education, marital history	Remaining years of life and proportion of remaining years with disability	USA		X	X	X								[49]
Microsimulation model	Race	Breast cancer mortality rate	USA	X	X	X	X					X		X	[50]
State-transition model	Race, gender	Life-expectancy	USA	X	X	X	X							X	[51]
State-transition simulation model	SES	Lung cancer incidence	UK	X	X		X		X					X	[52]
SIRS model	Region	Infectious disease transmission	(UK)	X	X	X					X			X	[53]
State-transition model	Education	Lung cancer incidence	Denmark	X	X		X		X					X	[54]
Dynamics systems	Region	Health, mortality	(Spain)	X	X						X			X	[55]
**Optimization**															
Optimal allocation model	Region	HIV prevention	USA	X							X	X	X	X	[56]
Location-allocation model	Region	Access to organ transplantation	Italy	X							X	X	X	X	[57]
Catchment population formulae	Region	Access to the health care system	Australia	X		X					X	X		X	[58]
Location-allocation model	Geographic location	Access to health services	India	X			X				X		X	X	[59]
**Optimization**															
Spatial interaction model	Region	Acute-care hospital utilization, accessibility	Australia	X	X	X					X			X	[60]
Spatial mathematical model	Region	Access to antiretrovirals	South Africa	X							X		X	X	[61]
Deterministic epidemic model	Province	Access to male circumcision	South Africa	X							X		X	X	[62]
Mathematical programming model	Program resources	Access to health care resources	(USA)	X									X	X	[63]
Goal programming model	Region	Nurses for maternal and child health services	China	X	X						X			X	[64]
Resource allocation formulae	Region	Patterns of health care delivery	UK	X							X		X	X	[65]
Formula for resource allocation	Local districts	Use of hospital services	Sweden	X		X	X				X			X	[66]
Resource allocation model	Zone of residence	Access to public service facilities	USA	X		X					X	X		X	[67]
Capacity-distance model	Commuting time	Access to dialysis	Japan	X			X		X		X		X	X	[68]
**Risk assessment**															
Stochastic multimedia exposure model	Region	Exposure to metals	France	X	X	X	X				X		X	X	[69]
Energy balance model	Income, poverty, education, ethnicity, geographic location	Exposition to heat stress	USA	X	X	X	X				X	X			[70]
**Risk assessment**															
Environmental equity rule	Ethnicity	Environmental risk on human health	USA	X							X			X	[71]
Source-receptor matrix	Geographic location	Premature death	USA	X		X					X		X	X	[72]
**Projection**															
Population projection model	Gender	Mortality, birth	China	X	X		X							X	[73]
Mathematical modelling	Geographic, economic sociocultural factors	Child mortality, stunting	14	X			X						X	X	[74]
**Game**															
Evolutionary variational inequality model	Perception of vaccine	Vaccination	(Canada)	X	X	X	X	X	X		X			X	[75]
Stackelberg game	Payment mechanism	Utilization of hospital services	Zambia	X										X	[76]
**Behavioral/stress**															
Behavioral model + decomposition	Social class based on occupation	Mortality, lifestyle	Great Britain	X	X	X	X		X		X				[77]
Stress model	Gender, education	Self-rated health status	any	X	X	X	X						X		[78]
**Diffusion**															
Mortality decline diffusion model	Geographic location	Mortality	(Israel)	X	X		X				X				[79]
